# Association between Lutein and Zeaxanthin Status and the Risk of Cataract: A Meta-Analysis

**DOI:** 10.3390/nu6010452

**Published:** 2014-01-22

**Authors:** Xiao-Hong Liu, Rong-Bin Yu, Rong Liu, Zhen-Xuan Hao, Cheng-Cheng Han, Zhong-Hai Zhu, Le Ma

**Affiliations:** 1 The First Affiliated Hospital, Xi’an Jiaotong University College of Medicine, 277 Yanta West Road, Xi’an 710061, China; E-Mail: liuxiaoh@mail.xjtu.edu.cn; 2 School of Public Health, Xi’an Jiaotong University College of Medicine, 76 Yanta West Road, Xi’an 710061, China; E-Mails: bloodwings198927@stu.xjtu.edu.cn (R.-B.Y.); Liu.rong@stu.xjtu.edu.cn (R.L.); hzxrjm@163.com (Z.-X.H.); hcc.89720@stu.xjtu.edu.cn (C.-C.H); zhuhai.47344250@stu.xjtu.edu.cn (Z.-H.Z.)

**Keywords:** lutein, zeaxanthin, blood, age-related cataract, meta-analysis

## Abstract

The purpose of this meta-analysis was to evaluate the relationship between blood lutein and zeaxanthin concentration and the risk of age-related cataract (ARC). MEDLINE, EMBASE, ISI and Cochrane Library were searched to identify relevant studies up to April 2013. Meta-analysis was conducted to obtain pooled relative risks (RRs) for the highest-versus-lowest categories of blood lutein and zeaxanthin concentrations. One cohort study and seven cross-sectional studies were included in the meta-analysis. There were significant inverse associations between nuclear cataract and blood lutein and zeaxanthin concentrations, with the pooled RRs ranging from 0.63 (95% confidence interval (CI): 0.49, 0.77) for zeaxanthin to 0.73 (95% CI: 0.59, 0.87) for lutein. A stronger association between nuclear cataract and blood zeaxanthin might be noted for the studies conducted in the European Nations. Blood lutein and zeaxanthin were also noted to lead towards a decrease in the risk of cortical cataract and subcapsular cataract; however, these pooled RRs were not statistically significant, with the exception of a marginal association between lutein and subcapsular cataract. Our results suggest that high blood lutein and zeaxanthin are significantly associated with a decrease in the risk of nuclear cataract. However, no significant associations were found for ARC in other regions of the lens.

## 1. Introduction

Cataract is a clouding or opacification of the lens inside the eye that obstructs the passage of light [1]. Age-related cataract (ARC) is the leading causes of blindness and vision impairment worldwide [2]. It was estimated that 20 million people older than 40 years were visually impaired due to ARC in the United States [3]. Although new therapeutic methods emerged in recent years and most ARC cases can be cured, the high treatment costs and increasing demands for therapy will challenge the long-term economic stability of health care systems [4]. With the rapidly aging population, ARC has brought a massive burden on health care and become an important public health issue. Thus, identifying modifiable factors available to prevent or delay the development of ARC is a crucial strategy.

Light-initiated oxidative damages are hypothesized to be the mechanism involved in ARC [5]. The xanthophyll carotenoids lutein and zeaxanthin are uniquely concentrated in the lens, where they can attenuate photochemical damage by filtering high-energy short-wavelength light [6,7]. In addition, they serve to protect the lens from oxidative damage by scavenging reactive oxygen species (ROS), indicating that these carotenoids may play a potentially important role in the prevention of ARC [8]. Numerous epidemiological studies have investigated the relationship between dietary intake and blood levels of lutein and zeaxanthin and the risk of ARC [9,10]. Because the accuracy of dietary intake measurements is greatly influenced by the different dietary assessment methods across the studies and the individual differences in utilization and absorption, blood concentrations appears to be a stronger predictor of nutritional status [11,12]. Many studies reported that high serum concentrations in lutein and zeaxanthin were inversely associated with the prevalence of ARC; however, others failed to find such association or the results regarding certain subtypes of ARC were inconsistent [13,14]. Therefore, we conducted this meta-analysis to evaluate the relationship between serum concentration of lutein and zeaxanthin and risk of difference ARC subtypes.

## 2. Experimental Section

This meta-analysis was performed in accordance with Preferred Reporting Items for Systematic Reviews and Meta-Analyses (PRISMA) guidelines [15].

### 2.1. Search Strategy

We searched MEDLINE, EMBASE, ISI Web of Science and Cochrane Library for relevant articles up to April 2013. Following MeSH words and text words were used: “lutein”, “zeaxanthin”, “xanthophyll”, or “carotenoid”, together with each of the following words “cataract”, “age-related cataract”, “ARC”, or “lens opacities”. Language restrictions were not imposed. Besides, manual searches of references cited by the retrieved articles were performed for additional literature. Authors and experts in the field were also contacted for relevant unpublished studies.

### 2.2. Study Selection

All retrieved articles were examined by scanning their titles and abstracts and any clearly irrelevant studies were excluded. Full texts of the remaining articles were further reviewed. Studies were included in this meta-analysis if they met the following criteria: (1) the primary outcome was clearly defined as ARC; (2) the exposure of interest was blood (plasma or serum) levels of lutein and zeaxanthin; (3) odds ratio (OR) or relative risk (RR) estimates and their 95% confidence intervals (CIs) were provided; and (4) potential confounders were controlled for by matching or multivariable analysis in the studies. If a series of articles were published from the same study, the report with the most updated data was selected for analysis. Literature search and screening were conducted independently by three investigators (RBY, RL, and ZXH) in a standardized manner, and any disagreements were resolved by discussion.

### 2.3. Data Abstraction and Quality Assessment

Information from eligible studies was abstracted independently by three investigators (X.H.L., R.B.Y., and R.L.), and discrepancies were resolved by consensus. We extracted the name of the first author, year of publication, country of origin, study design, characteristics of the study population (sample size, distribution of age, sex and ethnicity), diagnostic method of ARC, classification and grading systems for ARC, blood concentration assessment methods, fully adjusted odds ratio or relative risk, and the adjustment factors. If a study provided several risk estimates, the most completely adjusted estimate was extracted.

### 2.4. Statistical Analyses

For all studies, RRs or ORs and the corresponding 95% CIs were extracted from each article. Inverse variance-weighted risk estimates for ARC comparing the highest-versus-lowest categories of lutein and zeaxanthin were pooled with the fixed effects models or DerSimonian-Laird random effects models. The DerSimonian and Laird’s Q statistic and *I*² statistic were performed to evaluate statistical heterogeneity. Forest plots were used to visually assess the pooled ORs estimates across studies. Sensitivity analyses were performed to examine the influence of each individual study by iteratively excluding each study from the meta-analysis and comparing the point estimates including and excluding the study to determine their magnitude of influence on the overall estimate. Stratification and meta-regression analyses were performed to explore the source of heterogeneity among variables, such as country of origin, mean age of participants, method of ARC diagnosis and classification criteria of ARC. We assessed the potential of publication bias by using a funnel plot, Egger regression asymmetry test and Begg adjusted rank correlation test [16,17]. All statistical analyses were conducted by using Stata version 10.0 (Stata Corporation, College Station, TX, USA).

## 3. Results

### 3.1. Literature Search

**Figure 1:**
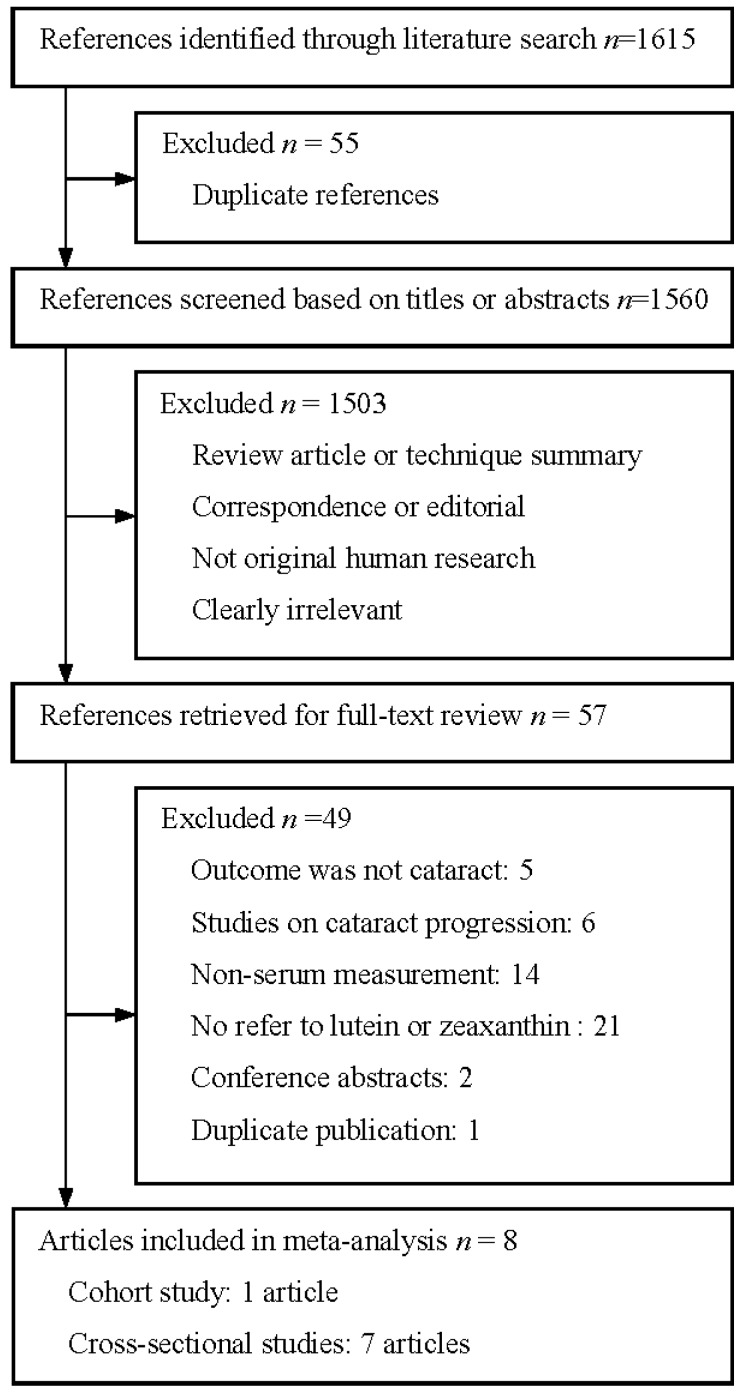
Flow diagram of study selection process.

Our search resulted in a total of 1615 references, of which 1558 were excluded after abstract review (Figure 1). Of the 57 articles that were considered as potentially relevant studies and were then retrieved for full-text review, eight studies (seven cross-sectional studies and one cohort study) met the inclusion criteria and were included in the meta-analysis [12,13,14,18,19,20,21,22].

### 3.2. Study Characteristics

Table 1 shows the design features and participant characteristics of the eight identified studies of blood lutein and zeaxanthin levels and risk of cataract. Of the included studies, three were conducted in the USA, three in Europe and two in India. Seven studies included both men and women, and one included only women. Cataract cases were ascertained based on lens photography in five studies, on slit lamp in two and on review of medical records in one. Four studies used the criteria of Lens Opacities Classification System (LOCS) to classify ARC, whereas the Early Treatment Diabetic Retinopathy Study (ETDRS) criteria and the Wisconsin Cataract Grading System (WCGS) criteria were applied in one study and two studies, respectively. In all of the included studies, levels of lutein and zeaxanthin were measured in blood samples by high-performance liquid chromatography (HPLC). All of the studies adjusted for age and smoking, six adjusted for gender and body mass index (BMI) and four adjusted for intake of alcohol. Other adjustment factors included serum cholesterol, education, iris color, blood pressure, social class, and history of diabetes.

### 3.3. Blood Lutein and Zeaxanthin and Nuclear Cataract

**Figure 2:**
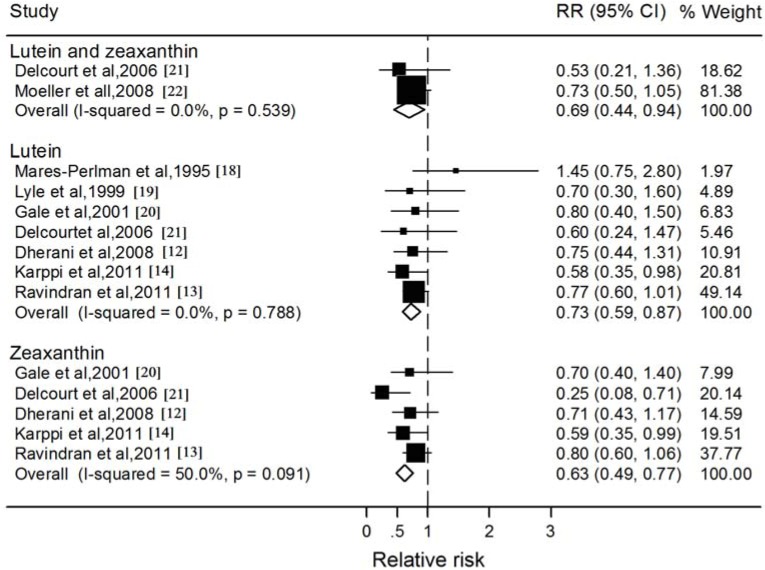
Forest plots of relative risks (RR) and 95% confidence interval (CI) for highest-*versus*-lowest category of blood concentrations of lutein and zeaxanthin and nuclear cataract risk.

Of the 8 studies included, two studies investigated the association between the blood lutein plus zeaxanthin and nuclear cataract, seven and five studies reported results for lutein and zeaxanthin, respectively (Figure 2). In all but one study, the RR for the highest category of blood lutein and zeaxanthin was associated with a decreased risk of nuclear cataract compared to the lowest category, ranging from 0.25 to 0.80. The pooled RR was statistically significant for blood lutein plus zeaxanthin concentration (RR: 0.69; 95% CI: 0.44, 0.94), and there was little evidence of statistical heterogeneity (*I*^2^: 0.0%; *P*: 0.54). For the individual xanthophyll carotenoids, the associations were consistently in the protective direction; however, blood zeaxanthin (RR: 0.63; 95% CI: 0.49, 0.77) tended to have a stronger association with decreasing the incidence of nuclear cataract than blood lutein (RR: 0.73; 95% CI: 0.59, 0.87). Exclusion of the cohort study essentially did not alter the results, with pooled RRs of 0.73 (95% CI: 0.59, 0.88) for lutein (Table 2). In addition, the stratified analyses showed consistent directions of effect when studies were grouped by the study and participant characteristics; however, the association between nuclear cataract and blood zeaxanthin was significantly stronger for the studies conducted in the European Nations (RR: 0.47; 95% CI: 0.26, 0.67) compared with those conducted in the India (RR: 0.78; 95% CI: 0.58, 0.97).

### 3.4. Blood Lutein and Zeaxanthin and Cortical Cataract

**Figure 3:**
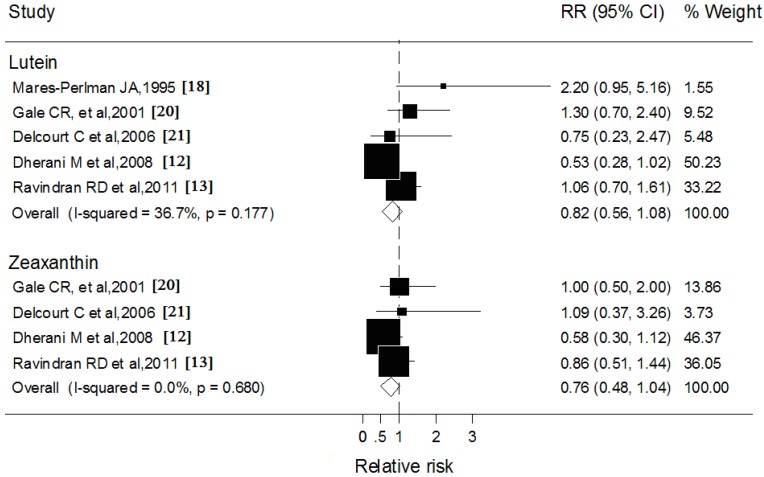
Forest plots of relative risks (RR) and 95% confidence interval (CI) for highest-*versus*-lowest category of blood concentrations of lutein and zeaxanthin and cortical cataract risk

Five studies reported data on blood lutein concentration with cortical cataract, and four studies reported blood zeaxanthin (Figure 3). The comparisons of the highest with the lowest blood concentrations of lutein (RR: 0.82; 95% CI: 0.56, 1.08) and zeaxanthin (RR: 0.76; 95% CI: 0.48, 1.04) were associated with 20% and 26% statistically nonsignificant reduction in cortical cataract risks, respectively. Results of subgroup analysis showed that none of the variables examined markedly influenced the shape of such association.

**Table 1:** Characteristics of studies of blood lutein and zeaxanthin and risk of age-related cataract.

Source, Year	Study Participants	Blood Assessment	Classification Criteria	Diagnosis Method	Controlled Variable
Mares-Perlman *et al.*, 1995 [18]	400 Americans aged 50–86 years in USA	HPLC	WCGS	Lens photography	Age, gender, smoking, alcohol intake and serum cholesterol
Lyle *et al.*, 1999 [19]	252 Americans aged over 50 years in USA	HPLC	WCGS	Lens Photograpuy	Age, smoking, alcohol intake, BMI and serum cholesterol
Gale *et al.*, 2001 [20]	372 Englishmen aged 66–75 years in England	HPLC	LOCS III	Slit lamp	Age, gender, smoking, alcohol intake, BMI, social class, serum cholesterol, glycosylated hemoglobin and steroids use
Delcourt *et al.*, 2006 [21]	815 Mediterranean people aged over 60 in France	HPLC	LOCS III	Slit lamp	Age, gender, smoking, education, iris color, plasma glutathione peroxidase, cardiovascular disease, diabetes, oral corticosteroids, cancer, and exposure to sunlight, *etc.*
Moeller *et al.*, 2008 [22]	1802 Americans aged 50–79 years in USA	HPLC	ETDRS	Lens photography	Age, smoking, BMI, iris color, physical activity, family history, multivitamin use, hormone replacement and pulse pressure
Dherani *et al.*, 2008 [12]	1112 North Indians aged over 50 years in India	HPLC	LOCS II	Lens photography	Age, gender, smoking, BMI, and systolic blood pressure
Ravindran *et al.*, 2011 [13]	5638 Indians aged over 60 years in India	HPLC	LOCS III	Lens photography	Age, gender, smoking, BMI, mid upper arm circumference, diastolic blood pressure, outdoor exposure, diabetes, current fuels, socioeconomic status, other antioxidants and study center
Karppi *et al.*, 2011 [14]	1689 Eastern Finnish aged 61–80 years in Finland	HPLC	Not mentioned	Medical records	Age, gender, BMI, smoking, alcohol intake, examination year, serum LDL-C, HDL-C, education, corticosteroids use, history of diabetes and hypertension with current use of antihypertensive medication

Abbreviations: BMI, body mass index; ETDRS, Early Treatment Diabetic Retinopathy Study; HDL-C, high density lipoprotein-cholesterol; HPLC, high-performance liquid chromatography; LDL-C, low density lipoprotein-cholesterol; LOCS, Lens Opacities Classification System; WCGS, Wisconsin Cataract Grading System.

**Table 2:** Stratified analysis of the association between serum level of lutein and zeaxanthin and nuclear cataract.

Subgroup	Numbers of Group	Pooled RR (95% CI)	*P* Value	
			Heterogeneity	Meta-Regression
**Lutein**				
Study design				
Cross-sectional studies	6	0.73 (0.59, 0.88)	0.68	0.92
Cohort studies	1	0.70 (0.30, 1.60)	NA	
Country of origin				
USA	2	0.92 (0.37, 1.46)	0.23	
Europe	3	0.63 (0.38, 0.88)	0.79	0.54
India	2	0.77 (0.58, 0.95)	0.94	
Mean age				
≤65 year	4	0.75 (0.58, 0.92)	0.96	0.71
>65 year	3	0.69 (0.44, 0.95)	0.26	
Method of diagnosis				
Lens Photography	5	0.77 (0.60, 0.94)	0.73	
Slit lamp	1	0.80 (0.40, 1.50)	NA	0.57
Medical records	1	0.58 (0.35, 0.98)	NA	
Classification criteria				
LOCS	4	0.76 (0.59, 0.93)	0.96	
WCGS	2	0.92 (0.37, 1.46)	0.23	0.50
Not mentioned	1	0.58 (0.35, 0.98)	NA	
**Zeaxanthin**				
Country of origin				
Europe	3	0.47 (0.26, 0.67)	0.20	0.03
India	2	0.78 (0.58, 0.97)	0.69	
Mean age				
≤65 year	3	0.63 (0.46, 0.80)	0.02	0.97
>65 year	2	0.62 (0.35, 0.89)	0.72	
Method of diagnosis				
Lens Photography	3	0.63 (0.46, 0.80)	0.02	
Slit lamp	1	0.70 (0.20, 1.20)	NA	0.94
Medical records	1	0.59 (0.35, 0.99)	NA	
Classification criteria				
LOCS	4	0.64 (0.48, 0.79)	0.05	0.80
Not mentioned	1	0.59 (0.35, 0.99)	NA	

Abbreviations: CI, confidence interval; LOCS, Lens Opacities Classification System; NA, not applicable because only one study; RR, relative risk; WCGS, Wisconsin Cataract Grading System.

### 3.5. Blood Lutein and Zeaxanthin and Subcapsular Cataract

**Figure 4:**
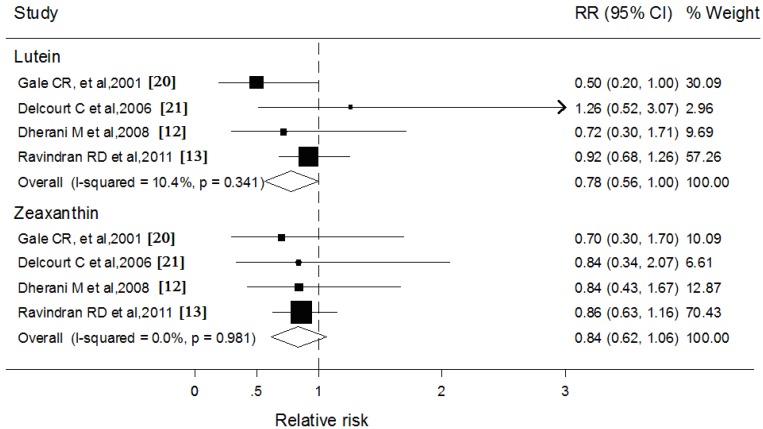
Forest plots of relative risks (RR) and 95% confidence interval (CI) for highest-*versus*-lowest category of blood concentrations of lutein and zeaxanthin and subcapsular cataract risk.

Four studies respectively reported blood levels of lutein and zeaxanthin (Figure 4). Pooled analyses showed no significant heterogeneity in the association between these two carotenoids and subcapsular cataract. Blood lutein was associated with a marginally significant reduction in the rate of subcapsular cataract (RR: 0.76; 95% CI: 0.56, 1.00), whereas the results for blood zeaxanthin were statistically nonsignificant (RR: 0.84; 95% CI: 0.62, 1.06). Similarly, inconsistencies in stratified variables also did not affect the overall pooled estimate for blood lutein and zeaxanthin and subcapsular cataract.

The funnel plots for the studies evaluating blood lutein and zeaxanthin concentrations and their association with different subtypes of ARC revealed symmetry. The Egger and Begg tests suggested the absence of significant publication bias (*P* > 0.05).

## 4. Discussion

The findings from this meta-analysis indicated that the reduction in the occurrence of nuclear cataract was significantly associated with high concentrations of lutein and zeaxanthin in serum, especially for zeaxanthin. Moreover, a stronger association between nuclear cataract and blood zeaxanthin might be noted for the studies conducted in the European Nations. However, no significant protective effects were found for each of these carotenoids against either cortical cataract or posterior subcapsular cataract, except a borderline significant association between blood lutein and subcapsular cataract.

The potential mechanisms for the positive effects of these carotenoids on ARC are not yet completely understood, but are thought to be primarily through their antioxidant properties and blue-light filtering activities [23]. The lens of the eye is particularly susceptible to oxidative damage; increased generation of ROS and high-energy short-wavelength light exposure leads to cross-linking or aggregation of the crystalline proteins in lens epithelial cells and thereby results in the generation of cataract [24]. Lutein and its isomer zeaxanthin are uniquely concentrated in the lens, indicating that each may have a possible particular function in this vital ocular tissue. They also have the potent antioxidant properties based on their abilities to quench singlet oxygen, scavenge superoxide and hydroxyl radicals, protect membrane phospholipids against UV-induced peroxidation, and reduce lipofuscin formation. In addition, absorption spectrum of these carotenoids peaks at 450 nm, consistent with the action spectrum for light-induced damage [25]. Moreover, lutein and zeaxanthin were incorporated in higher amounts into cell membranes in a single orientation, making them ideal optical filters. Therefore these carotenoids can absorb and attenuate the photic damage in the human lens [26].

Results from the present study indicated that blood concentration of lutein and zeaxanthin was significantly associated with the decreased risk of nuclear cataract. The consistency between our findings for each of these xanthophyll carotenoids indicated that such associations were robust. It should be noted that, compared to blood lutein, blood zeaxanthin showed a stronger inverse association with the occurrence of nuclear cataract. The seeming discrepancy was presumably explained by the difference in distribution pattern and biological properties of these two carotenoids. The ratio of zeaxanthin to lutein is much higher in human lens (0.8:1) than that in the plasma (0.2:1), suggesting that a selective uptake of zeaxanthin may occur in the lens tissue [27,28]. Moreover, zeaxanthin adopts a roughly perpendicular orientation with respect to the plane of the membrane, which makes zeaxanthin more effective in protecting the lens against the UV-induced membrane damage [29,30]. In addition, zeaxanthin appears to afford more protection to the liposomal lipids from light-induced oxidative stress, perhaps because zeaxanthin is a more effective singlet oxygen scavenger than lutein [31].

The stratified analyses showed that the association between nuclear cataract and blood zeaxanthin was significantly stronger for Europeans than it was for the Indians. This might be partly explained by the difference in zeaxanthin status for different populations. Compared with the Indians, blood zeaxanthin in Europeans was relatively higher [12,21,22]. Meanwhile, epidemiological data indicated that the average daily intake of lutein plus zeaxanthin was only about 2 mg per day and tended to less in Europe [32]. The consumption of these carotenoids was far below the level (above 6 mg per day) that had been associated with reduced risks of ARC [9]. Therefore, it is possible that Europeans with suboptimal zeaxanthin status might benefit more from improving blood zeaxanthin level via increasing consumption of foods rich in lutein and zeaxanthin. On the other hand, as the prevalence of the subtypes of ARC differs in different regions and populations, ethnic and racial differences might also have the potential to influence the association between nuclear cataract and zeaxanthin status [33].

With respect to cortical cataract and subcapsular cataract, we found no significant associations of these types of cataract with blood zeaxanthin or lutein. The difference in associations between cataract subtypes and serum zeaxanthin and lutein was probably due to the distinct pathogenesis for each type of ARC. With increasing age, a lower percentage of reduced glutathione can reach the lens nucleus, which makes the nucleus become less able to repair oxidative damage [34,35]. In contrast, glutathione levels in the outer cortex of the lens remain at high levels, even in nuclear cataracts [36]. Therefore, nuclear cataract may be more prone to a significant association with serum zeaxanthin and lutein. In addition, a marginally inverse relationship had been found between blood lutein and subcapsular cataract in the present study; however, subcapsular cataract is least common among these three main types of ARC and further studies are needed to confirm such finding [37].

The present study also has several potential limitations. First, our study is a meta-analysis of observational studies because of no randomized controlled trials (RCTs) found. It is commonly considered that observational studies are more likely to be subject to confounding and bias than RCTs, which can confound the results of our analysis. Second, the relatively small number of trials available evaluated the relationship between lutein and zeaxanthin status and the risk of cortical cataract or posterior subcapsular cataract; thus, our analysis was unlikely to detect substantially significant effect of xanthophyll carotenoids on opacities in these regions. Third, it may be that persons who have high blood levels of lutein and zeaxanthin are more likely to adhere to a healthier diet or lifestyle, which is associated with lower risks of ARC. Most studies adjusted for some major potential confounders; however, it was possible that residual or unknown confounding might bias the overall results in either direction. Fourth, although most subjects in studies from the European Nations were Europeans, it is possible that these study groups included participants of Asian and other non-European participants, which might lead to a mis-estimation of the association. Finally, although there was little evidence of publication bias from our funnel plot and rank correlation analysis, this possibility could be not completely excluded.

## 5. Conclusions

Our meta-analysis demonstrated that increased blood concentrations of lutein and zeaxanthin might be associated with a reduced risk of nuclear cataract. However, there is insufficient evidence to support a significantly inverse relationship between blood lutein or zeaxanthin level and risk of other subtypes of ARC. Meanwhile, there are only a few observational studies published about the association between blood concentrations of these carotenoids and ARC, which also limits the power of meta-analysis. Therefore, longer-term, large-scale, prospective controlled intervention studies are needed to clarify further the impact of lutein and zeaxanthin on the development of ARC.
